# Immigrant women’s experiences of maternity-care services in Canada: a protocol for systematic review using a narrative synthesis

**DOI:** 10.1186/2046-4053-1-27

**Published:** 2012-05-31

**Authors:** Gina M A Higginbottom, Myfanwy Morgan, Jayantha Dassanayake, Helgi Eyford, Mirande Alexandre, Yvonne Chiu, Joan Forgeron, Deb Kocay

**Affiliations:** 1 Faculty of Nursing, Edmonton Clinic Health Academy, University of Alberta, Edmonton, AB T6G 1C9, Canada; 2 King’s College London, Primary Care & Public Health Sciences London, London, SE1 3QD, UK; 3 School of Rural Health, Monash University, Bendigo, VIC, Australia; 4 Lois Hole Hospital, Alberta Health Services, Edmonton, AB, T5H 3V9, Canada; 5 Citizenship and Immigration Canada, New Multiculturalism Grants and Contributions Program, Canada Place, Edmonton, AB, T5J 4C3, Canada; 6 Multicultural Health Brokers Co-operative, Edmonton, AB, T5H 2M6, Canada; 7 Health Canada, Public Health Agency of Canada, Calgary, AB, T2G 4X3, Canada

**Keywords:** Narrative synthesis, Immigrant women, Maternity-care experiences, Canada, Study protocol, Mixed research design review

## Abstract

**Background:**

Canada’s diverse society and statutory commitment to multiculturalism means that the synthesis of knowledge related to the health care experiences of immigrants is essential to realize the health potential for future Canadians. Although concerns about the maternity experiences of immigrants in Canada are relatively new, recent national guidelines explicitly call for tailoring of services to user needs. We are therefore assessing the experiences of immigrant women in Canada accessing maternity-care services. We are focusing on: 1) accessibility and acceptability (as an important dimension of access) to maternity-care services as perceived and experienced by immigrant women, and 2) the birth and postnatal outcomes of these women.

**Methods:**

The aim of this study is to use a narrative synthesis, incorporating both a systematic review using narrative synthesis of reports of empirical research (qualitative, quantitative, and mixed-method designs), and a literature review of non-empirically based reports, both of which include ‘grey’ literature. The study aims to provide stakeholders with perspectives on maternity-care services as experienced by immigrant women. To achieve this, we are using integrated knowledge translation, partnering with key stakeholders to ensure topic relevancy and to tailor recommendations for effective translation into future policy and practice/programming. Two search phases and a three-stage selection process are being conducted (database search retrieved 1487 hits excluding duplicates) to provide evidence to contribute jointly to both the narrative synthesis and the non-empirical literature review. The narrative synthesis will be informed by the previous framework published in 2006 by Popay *et al*., using identified tools for each of its four elements. The non-empirical literature review will build upon the narrative-synthesis findings and/or identify omissions or gaps in the empirical research literature. The integrated knowledge translation plan will ensure that key messages are delivered in an audience-specific manner to optimize their effect on policy and practice change throughout the health service, and the public health, immigration and community sectors.

**Discussion:**

Narrative-synthesis methods of systematic review facilitate understanding and acknowledgement of the broader influences of theoretical and contextual variables, such as race, gender, socioeconomic status, and geographical location. They also enable understanding of the shaping of differences between reported outcomes and study designs related to childbearing populations, and the development and implementation of maternity services and health interventions across diverse settings.

**PROSPERO registration:**

Number 2185.

## Background

### Priority of health equity for multicultural Canada

In common with most western democracies, Canada is currently experiencing large-scale immigration and increasing ethnocultural diversity [1]. In fact, population growth over the past 100 years has largely resulted from immigration. Successive governments have pursued policies intended to actively encourage immigration and naturalization, thereby attempting to influence strategic demographic, economic, social, humanitarian, and security goals. Members of visible minority groups are expected to comprise between 29 % and 32 % of Canada’s population by 2031 [2].

The multicultural nature of Canadian society prioritizes the country’s commitment to equity in healthcare access and health outcomes. As in many other immigrant-receiving nations, the immigrants who enter Canada are relatively healthy, yet within 10 years of their arrival there is a convergence in terms of health status towards the Canadian average. A number of explanations have been suggested for this ‘healthy immigrant effect’ [3,4], including health selection [3]; acculturation; the stress of relocation, which may erode any health advantage [4]; and distrust of western medicine, with a preference for seeking out traditional healthcare providers. Furthermore, the needs and rights of immigrant women are often marginalized within families, communities, and legislation. Socioeconomic marginalization and the subsequent vulnerability of immigrant women can be further exacerbated by pregnancy and childbirth, making maternity an important focus of attention for those concerned with enhancing immigrant health.

### Perinatal health disadvantage for immigrants

Some studies (largely epidemiological), from Canada and elsewhere, report equal or more favorable birth outcomes for migrants [5-8] thus supporting an ‘epidemiological paradox’ associated with the concept of the ‘healthy immigrant effect’. These results imply that (selective) immigration from non-industrialized countries is associated with protective individual characteristics. Conversely, numerous reports highlight serious problems of equity in birth outcomes [9-11], particularly for refugees [12] and other immigrants after increased lengths of stay [13,14].

Our preliminary review established that one systematic review of immigrant-receiving countries in Europe found substantial disadvantages for immigrant as compared to natively born women in all of their outcomes: overall risks that were 43 % higher for low birth weight, 24 % higher for pre-term delivery, 50 % higher for perinatal mortality, and 61 % higher for congenital malformations [9]. A recent Canadian study found higher rates of low birth weight and full-term low birth weight (that is, small for gestational age (SGA)) for infants born to recent immigrant women [10]. The hospital costs for pre-term and SGA newborns are nine and two times higher, respectively, than those for their normal-growth counterparts [15]. Although these considerations are important, calculating birth outcomes (largely birth weight and infant mortality) is not an adequate or all-encompassing measure for evaluating maternal and infant health and well-being over their lifetimes.

Negative maternal characteristics and birth outcomes of immigrants include significantly higher rates of gestational diabetes (predisposing the mothers to pre-eclampsia and type 2 diabetes, and their offspring to obesity and type 2 diabetes) [16,17]; food proscriptions, resulting in low maternal weight gain (compromising both newborn and maternal health) [13]; genetic anomalies such as neural-tube defects due to lack of folic-acid intake [18]; and maternal anemia (increasing the risk of pre-term delivery) [19]. Successfully providing appropriate maternity care requires the legitimization and incorporation of the pervasive traditional beliefs and practices of immigrant women, which they often adhere to despite their new environment [20,21].

### Utilization of and access to health care by immigrant women

Conflicting evidence exists regarding underutilization/overutilization of health services by immigrant communities [22]. Some studies have reported that women may have more frequent contact with health services compared with men, because of their maternity needs [5]. Alternatively, migrant women have been reported to underutilize formal health care and other community services, largely because of language barriers, difficulties in understanding healthcare information, experiences of discrimination, and challenges in navigating the Canadian healthcare system [23,24]. Other explanations focus on socioeconomic position, including material disadvantage, geography, racial harassment, and exclusion [25-27].

Pregnant immigrant women may opt for obstetric rather than midwifery care during the prenatal and intrapartum period [28], and non-medical support may be important in terms of navigation of the healthcare system and also during the postnatal period. In some areas, a doula (non-medical labor coach) will provide important emotional support to immigrant women; these doulas are often unregistered midwives of immigrant background themselves. Recent research has found that migrant women often do not follow-up on referrals for postbirth care as suggested by community health nurses [29], and postnatal health concerns may not be addressed throughout provincial healthcare systems in Canada [30]. Moreover, the medicalized view of maternity promoted by western biomedicine may powerfully influence immigrant women’s perceptions of maternal care in ways that may not be congruent with their frames of reference. The international literature arising from Europe [31-33], Australia [34-36], and the US [37,38] demonstrates similar challenges for newcomer women.

### Knowledge gap regarding the maternity-care experiences of immigrants

Although a large-scale quantitative project, the Maternity Experiences Survey [28], was conducted in 2006 in Canada, the findings related to immigrant (and more so to refugee) women may be limited to some extent because of the survey being conducted only in English and French (the availability of a glossary of terms in various languages may or may not have proved useful), and over the telephone. Moreover, the cross-cultural validity of the survey was not evaluated [39]. Likewise, although an international research collaboration formed by Gagnon (McGill University) and Small (LaTrobe University, Australia), termed Reproductive Outcomes and Migration (ROAM) [40], has provided crucial insight into reproductive health (focusing on perinatal health) of migrants, the majority of its work was epidemiologically based without the specific focus on experiences of migrant women. Our knowledge synthesis will compliment this emerging body of research.

Performing a funded policy analysis and international comparison of maternity care for migrants in the UK, Canada and Germany, Salway *et al*. discovered that although there are contrasting policy and practice contexts, there are similar challenges in delivery of maternity care services in these countries, with underlying barriers and supports to good health outcomes for migrants [41]. Subsequently, Higginbottom *et al.* are currently using an interdisciplinary perspective to explore the maternity experiences of immigrant women in Alberta [42]. Additionally, during our preliminary database search and review of the Canadian empirical literature (as used to justify this systematic review) the evidence base strongly suggested that similar challenges exist for immigrant women in new host communities. Moreover, the Canadian evidence on this topic seems to arise from smaller scale, regional and provincial studies and we could not find any evidence of a published knowledge synthesis arising from the Canadian context.

## Methods and design

### Study aim and objectives

The aim of this study is to provide stakeholders with perspectives on maternity care services as experienced by immigrant women. This knowledge synthesis will use a systematic review with narrative synthesis of reports of empirical research and a literature review of non-empirically based reports, both incorporating ‘grey literature’. In this study, we assess the acceptability of relevant processes at the individual, community, and organizational levels, as these factors are recognized to be important determinants of effectiveness of services and patient/client outcomes. We identify specific crucial points in care delivery, providing tailored solutions for policy and practice changes. For the study, we use an approach known as ‘integrated knowledge translation’ (IKT) described as “KT (knowledge translation) woven into the research process” (Canadian Institutes of Health Research [43]), by partnering with key stakeholders (integrated knowledge users; IKUs) to ensure topic relevancy and to tailor our messages and recommendations to facilitate effective end-of-study KT (the exchange, synthesis and ethically-sound application of knowledge) for promoting translation into future policy and practice or programming. Partnerships with IKUs were started during the establishment of the research questions, will be fostered throughout the project, and have enabled early planning for dissemination. Using this IKT framework, this project will enable us to: 1) identify, appraise, and synthesize reports on empirical research using qualitative, quantitative, and mixed-method designs; 2) identify, appraise and synthesize reports and publications that do not have an empirical basis, and; 3) share our findings through strategic end-of-study KT to generate and disseminate important recommendations for future policy and practice/programming.

### Research question

The research question to be addressed is: what are the experiences of immigrant women in Canada accessing maternity-care services? Focus will be placed on: 1) accessibility and acceptability as an important dimension of access to maternity-care services, as perceived and experienced by immigrant women; and 2) birth and postnatal outcomes.

### Population of interest

This study will review literature and other documents that report on immigrants in Canada; we define an ‘immigrant’ as ‘a person who has settled permanently in another country (Canada)’ [44], but we also include economic migrants and skilled workers, temporary foreign workers, documented and undocumented residents, refugee claimants, refugees, asylum seekers, and students [45].

### Study design

This study will use three approaches that align with our objectives: 1) a systematic review with narrative synthesis, to identify, appraise and synthesize reports on empirical research using qualitative, quantitative and mixed-method designs; 2) a non-empirical literature review to identify, appraise and synthesize reports and publications without empirical basis, and; 3) IKT, for which we partner with stakeholders throughout the study and during end-of-study KT, to ensure topic relevancy, and to generate and disseminate important recommendations for future policy and practice/programming.

### Search strategies and selection of studies

The search and selection strategies draw upon established systematic review methods as outlined by the Centre for Reviews and Dissemination [46], and also incorporate recent guidelines for the selection, appraisal, and review of grey literature [47,48]. Grey literature is a field in library and information science that deals with the production, distribution, and access to multiple document types produced at all levels of government, academia, business, and organization, which are delivered in electronic and print formats not controlled by commercial publishing, and thus publishing is not the primary activity of the producing body [49]. Grey literature is not published commercially or indexed by major databases, although it can influence research, teaching, and learning [50]. Producers of grey literature report that policy-makers are their primary targeted audience, and three of the most important topic areas are access to health care, maternal and child health, and minority health [48].

**Figure 1:**
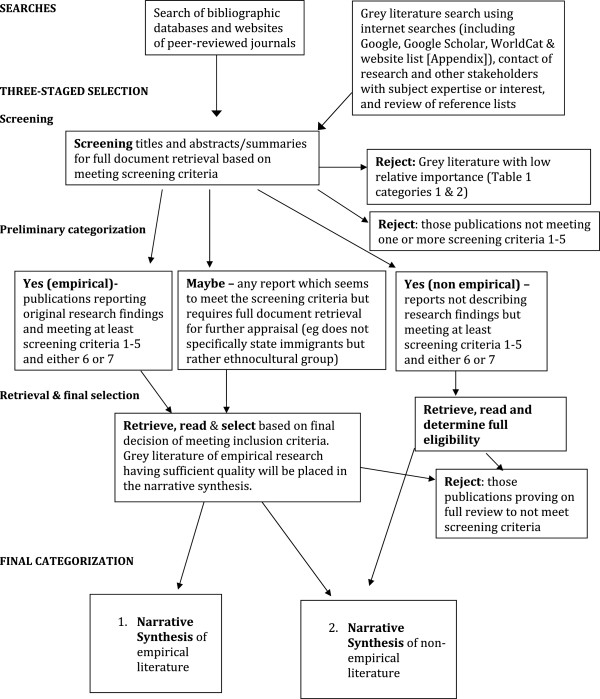
**Search and selection process.**. Two search phases will provide evidence to contribute jointly to both the narrative synthesis and literature review. The screening stage of selection (nearly complete) applies a checklist (Figure 2); the first five items and one of the last two items must be met to include the literature, which will be classified later as being empirical or non-empirical. Ambiguous items will be read in full and discussed by two or possibly three reviewers. Further selection or rejection will be completed after reading full reports and categorizing them as contributing to the narrative synthesis or literature review. Grey literature is being rejected at the screening stage if it is not considered to be of sufficient relative importance (Table 1, categories 1, 2 & 3) as determined by AcademyHealth [48]. Grey-literature reports describing empirical research will be placed in the narrative synthesis if deemed to have sufficient quality (category A, in accordance with McGrath *et al.*[47].

Two search phases are being conducted (with screening nearly complete) to provide evidence to contribute jointly to both the narrative synthesis (empirical evidence) and literature review (non-empirical publications in a variety of formats) (Figure 1). The search of electronic databases and websites of relevant journals largely retrieves empirical and non-empirical papers published in peer-reviewed journals. The search for grey literature is including select database searches (ProQuest Dissertations and Theses, Google, and Google Scholar), internet-based searches (see dditional file 1), review of reference lists, and email or phone contact with research and other stakeholders who have subject expertise or interest.

An information scientist (health research librarian) designed the database search strategies, which were reviewed by the entire research team, including IKUs, before implementation. The following databases have been searched: Ovid MEDLINE in-process and other non-indexed citations, Ovid MEDLINE daily, Ovid MEDLINE 1950 to present, Ovid PsycINFO 1987 to present, Ovid EMBASE 1980-present, EbscoHOST CINAHL 1937 to present, ISI Web of Knowledge Social Sciences Citation Index 1898 to present, ISI Web of Knowledge Sciences Citation Index 1899 to present, Scopus 1960 to present, and CSA Sociological Abstracts 1952 to present. The search strategy for Ovid MEDLINE (see Additional file 2) was reviewed and revised to use subject headings that are appropriate for the other databases. The results (1897 hits with 410 duplicates) from the implemented searches have been stored in RefWorks and have been screened and retrieved for selection (underway). There will also be hand searches within websites of relevant journals (such as *J Immigr Minor Health*, *JOGNN*, *J Health Serv Res Policy*, *Can J Public Health*, and *Culture, Health and Sexuality*) using key phrases and authors.

**Figure 2:**
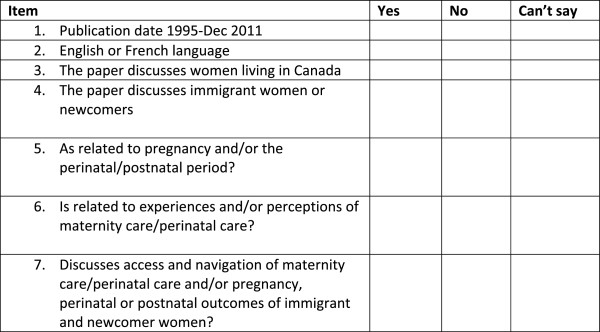
**Screening criteria checklist.** The first five items and one of the last two must be met for retention. Literature with ‘can’t say’ checks are being placed in the ‘maybe’ folder for full retrieval and further screening.

Selection of the studies for the narrative synthesis and non-empirical literature review is following a three-stage process of screening, preliminary categorization, and retrieval and final selection (Figure 1). The first process (complete for database search but not grey literature search) applies a screening criteria checklist (Figure 2), in which the first five items and one of the last two items must be met to allow classification as ‘yes, empirical’ (n = 63 items currently in this category) or ‘yes, non-empirical’ (n = 40). Literature that cannot be confirmed as meeting the screening criteria have been placed in a ‘maybe’ folder (n = 65) and retrieved in full for further screening. The full reports of all ‘yes’ and ‘maybe’ literature is being retrieved, and further selection or rejection will commence shortly. The final categorization will be made using the literature categorized as contributing to either the narrative synthesis (systematic review) or literature review of non-empirical literature. Study populations that are described solely as ‘ethnic minorities’ without mention of immigrant status or country of birth are not being considered because of the ambiguity inherent in the description. Systematic reviews are not being selected, although the reference lists of these papers will be reviewed for possible identification of further empirical literature. Additionally, grey literature will be rejected at the screening stage if it is not considered to be of sufficient relative importance (Table 1, categories 1, 2, & 3), as determined by the AcademyHealth Advisory Committee definition of the scope of grey literature in health services research/health policy for the National Information Center on Health Services Research and Health Care Technology at the National Library of Medicine) [48]. Reports seemingly of empirical research (using qualitative, quantitative, or mixed-methods research) derived from the database searches (largely published in peer-reviewed journals) will be placed in the narrative synthesis after confirmation of their empirical status (primary research using working hypotheses (or research questions), which are tested using observations or experimentation) [51], whereas all non-empirical publications will be used for the non-empirical literature review. Moreover, reports describing empirical research retrieved from the grey-literature search will be placed in the narrative synthesis if deemed to have sufficient quality (category A, in accordance with McGrath *et al*. [47]). Any grey literature deemed to have insufficient quality or empirical basis will be placed into the non-empirical literature review, because these publications will provide more for background or contextual information (category B, in accordance with McGrath *et al*. [47]). Grey literature passing the preliminary quality assessment will present a clear research question(s), state key findings, and provide sufficient details on the population(s) studied, interventions, study design, method of analysis, and evaluation outcomes [47]. Further quality appraisal will be conducted within the narrative-synthesis process. For the screening stage, one reviewer has screened all retrieved citations from the database searches, with those deemed questionable for selection (the ‘maybe’ folder) brought to one of the team leads (GH or MM) for a final decision. Concurrently, the entire team is engaging in a search of grey literature following an allocation of websites as decided collaboratively during a team meeting. Members have undergone adequate training by the information scientist on searching strategies for grey literature. In the final selection and categorization stages, there will be two investigators working independently, with any disagreement being resolved by one of the study leads (GH or MM). Inter-rater reliability of the grey literature quality assessments will be appraised, as will be the quality appraisal within the narrative synthesis (refer to element 4 of the narrative synthesis).

**Table 1:** **Relative importance of grey literature as used by AcademyHealth in their Grey Literature Project**[48]

**5**	**4**	**3**	**2**	**1**
Working papers	Data evaluations	Speeches	Newsletters	Pamphlets
Committee reports	Foundation reports	Annual reports	Biographies	Protocols
Testimony	Government reports	Presentations	Bulletins	Guidelines
Conference proceedings	Grantee publications	Grantee reports	Slide presentations	Poster sessions
	Non-commercially published conference papers			
	Reports	Webcasts	Foundation financial statements	Meeting agendas
	Special reports	Theses		Translations
		Technical specifications and standards		

Items were classified in categories based on a scale of frequency of use (5 most frequent ), and items in columns 5 and 4 were considered to have higher relative importance than the items in columns 3, 2 and 1, which will not be chosen for review in this study.

### Data extraction

For the narrative synthesis, a summary of each study reviewed will be tabulated using ATLAS.ti data analysis software (ATLAS.ti Scientific Software Development GmbH, Berlin, Germany), in which the lead author has training and consults as a trainer through an affiliation with the North American ATLAS.ti Training Center. Publications will be uploaded as primary documents (in portable document (PDF) format; Adobe Systems Inc., San Jose, CA, USA) and coded in accordance with the summary data requirements (considered as variables which can be imported from an Excel spreadsheet; Microsoft Corp., Redmond, WA, USA), and later with respect to thematic analysis. The review team will collectively decide on data variables (for example study design, sample population), bearing in mind those categories suggested as informative for textual descriptions within the narrative synthesis (Table 2; also refer to element 2 described in the section headed ‘Narrative Synthesis’). One reviewer will extract the data, and one or more team members will review for accuracy and completeness. The publications reviewed for the non-empirical literature review will generally be those other than research studies, hence narrative descriptions of the key messages of those publications will be constructed.

**Table 2:** **Tools of possible value for elements 2 to 4 of the narrative synthesis**[46,52]

	**Tasks**	**Explanation**
Element 2: preliminary synthesis of findings	Textual description of the studies	A descriptive paragraph with headings of ‘Setting’, ‘Participants’, ‘Aim’, ‘ Sampling and recruitment’, ‘Method’, ‘Analysis’, ‘Results’, and ‘thick’ or ‘thin’ study. This may be represented in tabular format
	Grouping and clustering of studies	The data extracted for the textual description will allow papers to be grouped and thus enable patterns between and within studies to be identified. This will be informed by the research questions. Data may be grouped by a particular feature (for example, location, method, ethnic groups, form of analysis, or main findings)
	Translating data: thematic analysis	To identify main or recurrent themes in findings
Element 3: exploring relationships within and between studies	Moderator variables and subgroup analysis	Identifying study characteristics that vary between studies, or sample (subgroup) characteristics that might help explain differences in findings
	Ideas webbing and concept mapping	‘Ideas webbing’ conceptualizes and explores connections between the findings reported in the review studies and often takes the form of a spider diagram. ‘Concept mapping’ links multiple pieces of information from individual studies, using diagrams and flow charts to construct a model with relevant key themes
	Qualitative case descriptions	Descriptions of outliers or exemplars of why particular results were found in the outcome studies
Element 4: assessing the robustness of the synthesis	Weight of evidence or validity assessment	To enable scoring of studies, quality checklists (to be determined still) will be used, and weighted scores will be applied after agreement of all researchers
	Critical reflection	Summary discussion, covering: 1) the synthesis methodology (focusing on the limitations and their possible effect on the results); 2) evidence used (quality, reliability, validity, and generalizability); 3) assumptions made; 4) discrepancies and uncertainties identified, and how discrepancies were dealt with; 5) areas where the evidence is weak or nonexistent; 6) possible areas for future research; and 7) discussion of the evidence, considering the ‘thick’ and ‘thin’ evidence and commenting upon similarities and/or differences between the various sources of evidence

*CASP* Critical Appraisal Skills Programme.

### Narrative synthesis

Our synthesis will use the approach to narrative synthesis described by Popay *et al*. (page 5, [52]), which is defined as ‘an approach to the systematic review and synthesis of findings from multiple studies that relies primarily on the use of words and text to summarise and explain the findings of the synthesis’. This approach is equally suitable for both quantitative and qualitative studies, as the emphasis is on an interpretive synthesis of the narrative findings of research rather than on performing a meta-analysis of the data. It will allow us to encompass the cross disciplinary and methodologically pluralistic natures of research in this topic area of experiences and outcomes of immigrant women in maternity.

The general framework for a narrative synthesis comprises four main elements: 1) development of a theory of how the intervention works, why, and for whom; 2) development of a preliminary synthesis of the findings of the included studies, 3) exploration of the relationships in the data; and 4) assessment of the robustness of the synthesis. Although represented linearly, the processes are not necessarily independent of each other, and the synthesis often takes an iterative approach. Within each element, there is a variety of tools and techniques that may be used, depending on the nature of the research evidence. Additionally, additional tools and techniques may be used where appropriate [46]. For instance, because this synthesis will very likely not synthesize intervention studies exclusively, a framework of maternity-care experiences including accessibility and outcomes will be created, rather than a theory of these, as suggested for element 1. Table 2 lists the tools that the team envisions using for this synthesis and below is a brief description of each element.

### Element 1: developing a theory

A theory will not play a large role in this synthesis which aims to largely explore experiences of immigrant women rather than implement any intervention with measurable endpoints and outcome measures. As stated above, after reading the studies we will develop a preliminary framework of the maternity-care experiences and outcomes of immigrant women, which we will use to interpret and understand our synthesis. This will probably be an iterative process with multiple revisions as we work through Elements 2 – 4.

### Element 2: developing a preliminary synthesis

The preliminary synthesis will provide an initial description and mapping of the results of all included research studies. The synthesis will be further evaluated by the entire team to identify contextual and methodological factors that may have influenced the published results. Interrogation of the preliminary synthesis will facilitate construction of explanations as to how and why maternity services may have been implemented or may have affected immigrant women of childbearing age in a particular manner. Through careful organization of results, cross literature comparisons are facilitated, allowing patterns to emerge regarding both the women’s experiences of maternity services, and the development and implementation of health services and health care interventions. For the textual descriptions of the studies, we have adapted the assessment of “thick” or “thin” papers using Roen et al.’s work [53] in a systematic review of the evidence about the implementation of the interventions to reduce unintentional injuries among children, which itself drew on the work of Denzin et al. [54] and may apply to both quantitative and qualitative narrative findings as the emphasis is on textual analysis of the narrative findings. This approach distinguishes between papers that offered greater explanatory insights into their outcome of interest from those that offered a limited insight. Thick papers: 1) provide a clear account of the process by which their findings were produced, including the sample, its selection and size with any limitations or bias noted, and clear methods of analysis and adjustment for confounding in statistical studies; and 2) present a developed and plausible interpretation of the analysis based on the data presented. By contrast, thin papers: 1) lack a clear account of the process by which their findings were produced, and 2) present an under-developed and weak interpretation of the analysis based on the data presented.

### Element 3: exploring relationships within and between studies

Patterns emerging from cross literature comparisons will be subjected to further rigorous evaluation to identify factors that may explain differences, including variances in women’s experiences, the effects of maternity interventions, and implementation of maternity services. This evaluation will contribute to the understanding about barriers and enablers that shape maternity services for immigrant women. Evaluation will be conducted of the relationships between study characteristics and reported findings and the ways in which these relationships may correspond with key aspects reported in other literature included in the literature review. Careful attention will be paid to the heterogeneity of research methods, methodological approaches, and population characteristics encompassed in the literature through the application of narrative methods, which are particularly suited to synthesizing such findings. Key tools used here are moderator variables and subgroup analysis, ideas webbing and concept mapping, and qualitative case descriptions (Table 2).

### Element 4: assessing the robustness of the synthesis

Key to ensuring the robustness of the synthesis is the methodological quality of key literature and the analytical methods used to develop the narrative synthesis. Research studies will be critically appraised using the Critical Skills Appraisal Programme [55] and weighted accordingly as defined by the study team (to be determined). The team will consider the types of evidence retrieved (that is, relative contribution from qualitative and quantitative designs) to make further decision on the use of a weight-of-evidence approach, such as that described by Gough [56]. Such an approach may not always be appropriate, especially in situations where insufficient information about the methodological quality of studies included in the review is available [57,58]. Within our critical reflection (Table 2), qualitative papers will be assessed in accordance with the principles of confirmability, transferability, credibility, and dependability described by Lincoln and Guba [59]. There will be a clear description of the potential sources of bias within the synthesis process itself.

### Non-empirical literature review

The non-empirical literature review of published literature will essentially be a generic review [60] to provide additional understanding of the experiences of maternity-care services by immigrant women in Canada. It may build upon the findings of the narrative synthesis and/or identify omissions or gaps in the empirical research literature. The importance of reviewing this work partly stems from the fact that some non-empirical reports published in peer-reviewed journals, such as correspondence pieces, can highlight unique aspects of the topic that are not empirically studied, owing to, for instance, sufficient sample population (as with patient safety incidents, for example). It also relates to the fact that some audiences, such as policy-makers and foundations, place a high priority on grey literature to gather their information [48]. The search and selection procedures defined above, will be comprehensive, but no appraisal of quality will be undertaken because of the non-empirical basis of the publications. If suitable, the review will incorporate some conceptual or thematic analysis. Explicit statements of the limitations of using literature that may have limited validity will be discussed.

### Integrated knowledge translation

Research projects that are successful in dissemination are those that respond to a topical issue, have national significance, and involve commitment from a team including experienced investigators working in a supportive environment [61]. IKUs from various sectors are involved in this project to ensure the research is of contextual relevance for current policy, practice, and programming needs in Canada. The organizations represented include Citizenship and Immigration Canada (CIC), Public Health Agency of Canada (PHAC), Alberta Health Services (AHS), and the Multicultural Health Brokers Co-operative (MCHB Co-op). The CIC Multiculturalism and Human Rights Program (author ME is Program Director) promotes multiculturalism and community development by planning and organizing federal and community initiatives and programs, and by providing strategic advice about program policy and delivery to senior management both regionally and nationally. The PHAC (author DK is Program Consultant) funds the Community Action Program for Children and Canada Prenatal Nutrition Program, the latter providing long-term funding to community groups to develop or enhance programs for vulnerable pregnant women. The authors from AHS (HE and JF) represent the aspects of diversity education/strategy and nursing education of the provincial health system in one of Canada’s provinces. The MCHB Co-op is a unique workers’ co-operative that provides culturally respectful and responsive health education and outreach support to immigrant and refugee families in 17 immigrant and refugee communities. Current funded projects/programs include the Peri-Natal Outreach and Support program, the Home Visitation Program, the Multicultural Family Connections Program, and Interventions to Support and Provide Childcare Services to Refugee and Immigrant Families. Several authors (YC, ME and HE) have substantial contacts within immigrant support and/or settlement agencies throughout Canada. In addition to using IKUs, a systematic review expert (MM) will help ensure the rigor and validity of the findings and recommendations.

Although concern with immigrant maternity experiences is relatively new in Canada [62] recent national guidelines from the PHAC explicitly refer to population diversity and the need to tailor services to the needs of those they serve [28]. Furthermore, although not universal throughout Canada, voluntary sector immigrant agencies are active in several cities (for example the MCHB Co-op in Edmonton), supporting immigrant women in access to maternity services. A rapidly changing demography and observations from maternity-care providers throughout AHS, coupled with the known health disparities that immigrant women experience, present many challenges on the trajectory of maternity-care provision whether in the community or hospital.

### Partnership interactions

Interactions of the team have occurred during the preparation of this proposal to enable refinement of the research question, to optimize its relevancy for the entire team, and to identify potential strategies for dissemination. After study commencement, the entire team, including the information scientist, will meet to refine the search strategy, develop lists of websites and contacts for identifying grey literature, plan for future meetings, and identify KT opportunities. During the review, monthly scheduled meetings (and unscheduled meetings at crucial junctures) will occur, initially to reach consensus on final inclusion of studies, and, later, to discuss results, mainly related to grouping findings, exploring relationships, and determining themes. Once preliminary findings have been synthesized, the team will discuss the nature and potential significance of the emerging synthesis, to offer interpretation of the emerging themes, and to begin to devise recommendations and messages to target specific audiences. Subsequently, a specific pre-dissemination meeting will enable review of the significance of the findings, the dissemination activities, and the key messages. To this meeting we will invite other key decision-makers and practitioner colleagues identified by IKUs through their extensive links. Moreover, IKUs will help to identify, plan, and present dissemination events (including community-based events and conferences and symposia), and will assist with development and dissemination of other KT outputs such as the briefing paper, fact sheet, and seminars.

### Dissemination of findings and recommendations

Our dissemination goals are two-fold: 1) To ensure that key messages are delivered in an audience-specific manner, aligning with the needs of IKUs, to optimize their effect on policy and practice change throughout the health service, and the public health, immigration and community sectors; and 2) to utilize widely accessible technology (webinars and possibly social networking) to ensure maximum coverage. The specific KT strategies can be categorized by the target audience.

For mixed-audience KT, a research briefing paper for health practitioners, policy-makers and decision-makers in Canada and an accessible plain-language fact sheet will be professionally designed and produced, with wide dissemination using the activities and networks of academic team member and IKUs The fact sheets, in particular, will facilitate transfer of messages to the public and healthcare professionals, policy-makers and other knowledge users. Both will utilize the expertise of two of the IKUs (HE and ME) and will be linked with an electronic version. Contribution to academic theory and practice will occur through publication of findings in accessible international journals. The IKUs will be invited to co-author publications. A major portion of our efforts are also anticipated to be targeted towards PHAC and CIC to recommend revision of existing resources for women (for example, the Healthy Pregnancy Calendar and *The Sensible Guide to a Healthy Pregnancy*) to incorporate our findings (for example using language translation and incorporation of culturally appropriate concepts).

Given that the research team involves people who are engaged in community and hospital-based health services with immigrant women, knowledge translation has begun and will continue through to public dissemination through community meetings with families, women’s groups, and workshops related to refugee/immigrant health. Likewise, additional knowledge users (multiprovincial) will be invited to attend community-based seminars/workshops. Publicizing our findings at multicultural and immigration/multicultural events will target community, provincial, and national leaders, and two of the IKUs (YC and ME) will be primary agents for these activities.

Mechanisms for diffusion and transfer of knowledge will inform policy and practice, and will include international, national, and regional networks and conferences attended by policy-makers such as the National Metropolis Conference and the Qualitative Research for Policy Making Conference (UK); a multiprovincial electronic seminar; and a provincial email knowledge-transfer network called Collaborative Research in Ethnicity, Social Care, and Health, which will be created and convened by the first author (GH). Presentations will be made by the IKUs when they attend conferences and advisory committee meetings with key federal and provincial policy-makers and ministers.

An electronic seminar will be developed with presentations by the first author (GH) and IKUs to disseminate the findings to healthcare practitioners, and additional workshops/seminars may be facilitated at national health research conferences (for example, for neonatal and obstetrical nursing). Two other IKUs (JF and HE) will integrate findings into professional practice via their clinical and diversity education capacities.

## Discussion

A knowledge synthesis of maternity care among immigrants to Canada is needed to build a coherent evidence base eliciting understanding of factors generating disparities in accessibility, acceptability, and outcomes during maternity care, and to improve culturally based competency of maternity care. Synthesized evidence is needed in order for knowledge users within multiple sectors to configure maternity services and programs appropriately. In addition, synthesized evidence is needed for strategic enhancements to the current maternity-service provision, including professional development of health professionals to ensure provision of culturally congruent and culturally safe maternity care. Ultimately, enhancements to maternity care for immigrant women benefit not only the female service users but also the health of future generations of Canadians.

The lack of an available synthesis of this complex issue makes our knowledge synthesis significant and important as a mechanism for drawing together messages from research to guide policy and practice in meaningful ways. The heterogeneous nature of the methodological approaches to inquiry, being quantitative, qualitative and mixed-method approaches, mitigates against a meta-ethnographical approach or meta-data analysis, and supports our systematic review using a narrative synthesis. Moreover, narrative methods facilitate understanding and acknowledgement of the broader influences of theoretical and contextual variables, such as race, gender, socioeconomic status, and geographical location. They also enable understanding about the shaping of differences between reported outcomes and study designs in relation to childbearing populations and the development and implementation of maternity services and health interventions across diverse settings. The additional methods used to supplement and contextualize the narrative synthesis findings by identifying and analyzing non-empirical reports will enrich our understanding and dissemination of the findings.

## Abbreviations

IKT: integrated knowledge translation; KT: knowledge translation; IKU: integrated knowledge users; PHAC: Public Health Agency of Canada; CIC: Citizenship and Immigration Canada; MCHB Co-op: Multicultural Health Brokers Co-operative; AHS: Alberta Health Services.

## Competing interests

The authors declare no competing interests with this research.

## Authors’ contributions

GH conceptualized the study and prepared the draft of the research proposal. MM provided insight to the methodological approach of the narrative synthesis and quality appraisal. MM, JD, HE, ME, DK, YC and JF contributed to the proposal development as related to research questions, aim/objectives, and dissemination activities. All authors assisted with manuscript preparation and approve of the final submission.

## Supplementary Material

Additional file 1 List of websites generated for searching grey literature. Click here for file

Additional file 2 Search strategy overview and table for MEDLINE. Click here for file
