# Supplementary material for: Immigrant women’s experiences of maternity-care services in Canada: a protocol for systematic review using a narrative synthesis
Source: Syst Rev. 2012 May 31;1:27. doi: 10.1186/2046-4053-1-27 (PMC3433387; doi:10.1186/2046-4053-1-27)
Supplement: Additional file 1 — List of websites generated for searching grey literature. [file 2046-4053-1-27-S1.doc]

**Additional file 1: List of websites generated for searching grey literature**

During searches will identify search terms used and outcomes as relates to 1. Searched, nothing found, 2. Searched, relevant items found, 3. Not searched, 4. Searched items may be of relevance for background

**Clinical practice guidelines sites**

1. Canadian Medical Association. CMA Infobase.

<http://www.cma.ca/index.php/ci_id/54316/la_id/1.htm>

1. Ontario Guidelines Advisory Committee. (GAC) <http://www.gacguidelines.ca/index.cfm?pagepath=GAC_Endorsed_Guidelines&id=21080>
2. British Columbia Ministry of Health Services

<http://www.bcguidelines.ca/gpac/alphabetical.html>

1. Alberta Medical Association Towards Optimized Practice <http://www.topalbertadoctors.org/>

**Women’s health**

1. Mother and Child health Research (Aus) [www.latrobe.edu.au/mchr/](http://www.latrobe.edu.au/mchr/)
2. Centre for Research on Educational and Community Services: Ontario's Maternal, Newborn [www.rrasp-phirn.ca](http://www.rrasp-phirn.ca/) [Cached](http://webcache.googleusercontent.com/search?q=cache:JtQDVQ94-wYJ:www.latrobe.edu.au/mchr/+mother+and+child+health+research+la+trobe+university&cd=1&hl=en&ct=clnk&gl=au) - [Similar](http://www.google.com.au/search?hl=en&biw=1680&bih=853&q=related:www.latrobe.edu.au/mchr/+mother+and+child+health+research+la+trobe+university&tbo=1&sa=X&ei=ygG7TrfIAuXMmAXx-py6Dg&sqi=2&ved=0CCEQHzAA)Not helpful? You can block www.latrobe.edu.au results when you're signed in to search.www.latrobe.edu.au
3. Best start <http://www.beststart.org/> and <http://www.beststart.org/index_eng.html> and <http://www.beststart.org/resources/rep_health/index.html>
4. Health Canada’s women’s health pubs <http://www.hc-sc.gc.ca/hl-vs/pubs/women-femmes/index-eng.php>
5. The Source: Women’s Health Data Directory: Provides links and annotations to data sources of women’s health. Funded by Canadian federal and provincial government organizations: <http://www.womenshealthdata.ca/advancedsearch/default.aspx?rt=2>
6. <http://www.womenandhealthcarereform.ca/>
7. <http://www.motherhoodinitiative.org/>
8. Canadian women’s studies assoc <http://www.yorku.ca/cwsaacef/>
9. <http://thesurvey.womenshealthdata.ca/>

The Survey/Le Sondage is a bilingual web based repository that links women’s health practitioners, policy makers, health authorities, and women’s groups to a range of resources on women’s health, including international, national, and provincial documents. Materials such as local health authority reports, reports commissioned by governments, and those produced by and for community groups and citizens are included. The Survey/Le Sondage allows users to search for specific reports and other sources of grey literature that are pertinent to both the geographic area and topic of interest.

1. Canadian women’s health network <http://www.cwhn.ca/en>
2. Centres for excellence for women’s health <http://www.cewh-cesf.ca/en/index.shtml> database <http://www.cewh-cesf.ca/en/search.shtml> and research bulletin <http://www.cewh-cesf.ca/en/publications/RB/index.shtml>
3. Canadian foundation for women’s health <http://www.cfwh.org/>
4. SOGC Women’s Health Information <http://www.sogc.org/health/index_e.asp>
5. Women’s Health Research Foundation of Canada <http://www.whrfcinc.com/>
6. Women’s college hospital <http://www.wchospital.ca/>
7. Women and health protection <http://www.whp-apsf.ca/en/index.html>
8. Ontario’s women’s health network
9. The Source: women’s health data directory <http://www.womenshealthdata.ca/resources/>
10. Women’s health research network <http://www.whrn.ca/opportunities.html>
11. Canadian association of women’s health and perinatal nurses <http://www.capwhn.ca/en/capwhn/Home_Page_p2469.html>
12. Women’s health in women’s hands (Ontario health services site) <http://www.whiwh.com/>

**Immigrant/multicultural women’s health/associations**

1. Immigrant Women’s Health Centre (Toronto) <http://immigranthealth.info/>
2. Immigrant Women’s Centre (Ontario) <http://www.stjosephwomen.on.ca/>
3. Migration and reproductive health <http://migrationandreproductivehealth.org/?page_id=13>
4. South Asian Women’s Community Centre <http://www.sawcc-ccfsa.ca/EN/>
5. Indo-Canadian Women’s Association <http://www.icwaedmonton.com/>
6. South Community Birth program <http://scbp.ca/helpful-resources.html>
7. Calgary Immigrant Women’s Association <http://www.ciwa-online.com/>

**Provincial/federal health care & policy sites**

1. Status of Women of Canada <http://www.swc-cfc.gc.ca/index-eng.html>
2. Canada Prenatal Nutrition Program <http://www.phac-aspc.gc.ca/hp-ps/dca-dea/prog-ini/cpnp-pcnp/about-apropos-eng.php>
3. Canadian Public Health Association <http://www.cpha.ca/en/default.aspx>
4. Health Evidence <http://health-evidence.ca/>
5. Public Health Research, Education and Development <http://www.phred-redsp.on.ca/>
6. Health Canada – migrant health <http://www.hc-sc.gc.ca/sr-sr/pubs/hpr-rpms/bull/2010-health-sante-migr/index-eng.php> and health policy research bulletins <http://www.hc-sc.gc.ca/sr-sr/pubs/hpr-rpms/index-eng.php>
7. CHNET-WORKS Population Health Improvement Network <http://www.chnet-works.ca/>
8. Gov Canada Policy Horizons Canada (Policy research initiative) <http://www.horizons.gc.ca/homepage.asp?pagenm=root&langcd=E>
9. Canadian Association of midwives <http://www.canadianmidwives.org/resources.html>
10. Access alliance <http://accessalliance.ca/content/endorse-right-health-care-coalition>
11. Canadian Research Institute for the Advancement of Women <http://criaw-icref.ca/womenhealthandaction> <http://criaw-icref.ca/>
12. Child and Family Research Institute <http://www.cfri.ca/>
13. Alberta Health and Wellness: The Alberta Government Ministry of Health website, with ministry publications and a list of manuals and guidelines for health professionals: [http://www.health.gov.ab.ca](http://www.health.gov.ab.ca/)
14. British Columbia Centre for Health Services and Policy Research: Includes reports of various policy topics and is the repository of the former BC Office of Health Technology Assessment (BCOHTA): <http://www.chspr.ubc.ca/publications>
15. Effective Public Health Practice Project (EPHPP): Systematic reviews in public health produced by the Public Health Research, Education and Development (PHRED) Program, in Ontario: <http://old.hamilton.ca/phcs/ephpp/ReviewsPortal.asp>
16. Institute for Clinical and Evaluative Sciences (ICES): Ontario’s health services and utilization research agency: <http://www.ices.on.ca/>
17. Manitoba Centre for Health Policy (MCHP):Manitoba’s health services and utilization research agency. Source of administrative data: <http://umanitoba.ca/medicine/units/mchp>
18. Gov of Canada – Policy Research Initiative

<http://www.policyresearch.gc.ca/homepage.asp?pagenm=root&langcd=E>

**Canadian immigration/multicultural sites**

1. Changing together (a centre for immigrant women) <http://www.changingtogether.com/>
2. Canadian heritage <http://www.pch.gc.ca/eng/1266037002102/1265993639778>
3. Citizenship and Immigration Canada <http://www.cic.gc.ca/english/index.asp>
4. Canadian Council of refuges links <http://ccrweb.ca/en/links>
5. Manitoba Gov’s Labour and Immigration <http://www.gov.mb.ca/msw/resources/links.html>
6. Canada Updates (Making immigration simpler) <http://www.canadaupdates.com/> <http://www.canadaupdates.com/content/rate-premature-birth-high-among-immigrant-women-spending-years-canada-research-16399.html>
7. National Organization of Immigrant and Visible Minority Women of Canada <http://www.noivmwc.org/>
8. Metropolis <http://canada.metropolis.net/>

**Health information**

1. Canadian Institute for Health Information (CIHI) <http://www.cihi.ca/>
2. New Brunswick Ministry of Health Epidemiological Service. Health Publications and Services in New Brunswick [http://www.gnb.ca/0051/pub/index-e.asp#epidemiology](http://www.gnb.ca/0051/pub/index-e.asp#epidemiology)
3. Public Health Agency of Canada Reports & Publications (PHAC)

<http://www.phac-aspc.gc.ca/publications-eng.php>

1. PHAC Surveillance (PHAC) <http://www.phac-aspc.gc.ca/surveillance-eng.php>
2. Ontario Health Coalition <http://www.web.net/~ohc/>
3. Canadian Academy of Health Sciences <http://www.cahs-acss.ca/>
4. Canadian Health Services Research foundation <http://www.chsrf.ca/Splash.aspx>

**Immigrant Serving Organizations**

1. Integration Net (by CIC) <http://integration-net.ca/>
2. Welcoming Communities <http://welcomingcommunities.ca/>
3. Ontario Community Immigrant Services Association <http://ociso.org/En/>
4. Alberta Association of immigrant Service Agencies <http://www.aaisa.ca/>
5. Mosaic BC <http://www.mosaicbc.com/family-programs>
6. Welcome BC <http://www.welcomebc.ca/wbc/service_providers/programs/settlement_agencies.page>
7. AMSSA <http://www.amssa.org/>
8. Ontario’s Council on Agencies Serving Immigrants <http://www.ocasi.org/index.php?qid=785&catid=115>
9. Canadian Newcomer Magazine <http://www.cnmag.ca/>

**Cultural organizations in Alberta**

1. Catholic Social Services <http://www.catholicsocialservices.ab.ca/CatholicSocialServices/default.aspx>
2. Edmonton Mennonite Centre <http://www.emcn.ab.ca/>

**International Health Evidence sites**

1. Health Evidence Network (HEN): A WHO site that provides assessments of public health interventions for health care decision-makers: <http://www.euro.who.int/en/what-we-do/data-and-evidence/health-evidence-network-hen>
2. CEBM http://www.cebm.net/

**Databases (Google and Google Scholar will also be searched)**

1. CRD databases <http://www.crd.york.ac.uk/crdweb/>
2. Campbell Collaboration Library of Systematic Reviews <http://www.crd.york.ac.uk/crdweb/>
3. Health-Evidence.ca: Searchable online database of systematic reviews of public health and health promotion interventions geared towards decision makers: <http://health-evidence.ca/articles/search>
4. Evidence for Policy and Practice Information and Coordinating Centre (EPPI-Centre): provides 3 databases useful in the area of health promotion:
   - The Trials Register of Promoting Health Interventions (TRoPHI): Database of citations to health promotion-related studies and trials literature <http://eppi.ioe.ac.uk/webdatabases/Intro.aspx?ID=5> .
   - Bibliomap: the EPPI-Centre database of health promotion research. All studies are coded for specific characteristics of health focus, population group and study type: <http://eppi.ioe.ac.uk/webdatabases/Intro.aspx?ID=7> .
   - Database of Promoting Health Effectiveness Reviews (DoPHER)- database of health promotion reviews (mostly systematic reviews). Updated quarterly: <http://eppi.ioe.ac.uk/webdatabases/Intro.aspx?ID=2> .
5. ERIC: Database of the Educational Resources Information Center includes health-related educational information: [http://eric.ed.gov](http://eric.ed.gov/)
6. Social Policy & Practice Database <http://bathhealthnews.blogspot.com/2009/11/new-database-social-policy-practice.html>

**Library catalogue and book databases**

1. Amicus, The Canadian National Catalogue: Includes all titles in the National Library and many other Canadian libraries. *Tip: If you have a complex search, quickly create an account and log in using the registered service so that you can do command searching*: <http://www.collectionscanada.ca/amicus> .
2. Theses Canada: A central access point for Canadian theses. Full text of digitized theses and dissertations are available from 1998 on: <http://www.collectionscanada.gc.ca/thesescanada/>
3. WorldCat: Includes over 1.4 billion items from more than 60,000 libraries worldwide. Useful to discover if the library near you has the book you are looking for: [http://www.worldcat.org](http://www.worldcat.org/)
4. Amazon.com: Although a commercial enterprise, Amazon is also a very large book database that is a useful tool to discover the existence of books on a particular subject. Searching is free and you might be able to find the book at your local library or through interlibrary loan: [http://amazon.com](http://amazon.com/)

**Statistics**

1. Database of Online Health Statistics: Compiled by the information specialists at the Institute of Health Economics, this database provides quick and easy access to freely available web-based statistics generated by national and global agencies and research groups: <http://www.ihe.ca/publications/health-db>
2. Statistics Canada: Canada’s national statistical agency. All electronic reports are available free of charge as of April 2006: [http://www.statcan.gc.ca](http://www.statcan.gc.ca/)

**Health economics sites**

1. Centre for Health Economics, University of York: Research at the Centre includes economic assessments of health technologies: <http://www.york.ac.uk/inst/che> .
2. Centre for Health Economics and Policy Analysis (CHEPA), McMaster University: Working paper publications include many relevant to economic evaluation and health technology assessment: [http://www.chepa.org](http://www.chepa.org/)
3. Institute of Health Economics (IHE): Canadian research in health economics, outcomes, policy research, and health technology assessment: [http://www.ihe.ca](http://www.ihe.ca/)
